# Detection of circulating BMP5 as a risk factor for Barrett’s esophagus

**DOI:** 10.1038/s41598-020-70760-1

**Published:** 2020-09-23

**Authors:** Ana C. P. Correia, Silvia Calpe, Nahid Mostafavi, Sanne Johanna Maria Hoefnagel, Maria del Carmen Sancho-Serra, Patricia S. de Koning, Kausilia K. Krishnadath

**Affiliations:** 1 Center for Experimental and Molecular Medicine (CEMM), Amsterdam University Medical Center, Location AMC, Amsterdam, The Netherlands; 2 Department of Gastroenterology and Hepatology, Amsterdam University Medical Centers, Location AMC, Amsterdam, The Netherlands; 3 Department of Gastroenterology and Hepatology, Subdivision Statistics, Amsterdam University Medical Centers, Location AMC, Amsterdam, The Netherlands; 4 Present Address: Sanger Institute, Wellcome Trust Genome Campus, Hinxton, Cambridge, UK

**Keywords:** Biomarkers, Gastroenterology, Medical research

## Abstract

Barrett’s esophagus (BE) predisposes for the malignant condition of esophageal adenocarcinoma (EAC). Since BE patients have few or no symptoms, most of these patients are not identified and not included in surveillance programs. These BE patients are at risk of developing advanced-stage EAC. At present, non-invasive tests to identify BE patients from the general population are lacking. We and others showed that Bone Morphogenetic Protein 4 (BMP4), and other BMPs are upregulated in BE. We aimed to determine if circulating BMPs can be identified and used as blood biomarkers to identify BE patients at high risk in the general population. In this study, we could detect the different BMPs in the blood of 112 BE patients and 134 age- and sex-matched controls. Concentration levels of BMP2, BMP4, and BMP5 were elevated in BE patients, with BMP2 and BMP5 significantly increased. BMP5 remained significant after multivariate analysis and was associated with an increased risk for BE with an OR of 1.49 (*p* value 0.01). Per log (pg/mL) of BMP5, the odds of having BE increased by 50%. Future optimization and validation studies might be needed to prove its utility as a non-invasive method for the detection of BE in high-risk populations and screening programs.

## Introduction

Barrett’s esophagus (BE) is a premalignant condition in which the normal epithelial squamous mucosa of the distal esophagus acquires an intestinal columnar phenotype^1,2^. This metaplastic condition has clinical importance due to its association with an increased risk of esophageal adenocarcinoma (EAC)^3^. The prevalence of BE is around 1–5% in Western populations^1,4^, with a yearly conversion rate for EAC between 0.1 and 0.9% in non-dysplastic BE patients^5–7^, 0.5–3% in patients with low-grade dysplasia (LGD)^8,9^, and 7–13.4% in patients with high-grade dysplasia (HGD)^10–12^. The overall 5-year survival rate of EAC is lower than 20%^5,7,13^.

Patients diagnosed with BE are included in endoscopic surveillance programs and subjected to periodic follow-up endoscopies for detection and treatment of EAC in the early stage^1^. As this can improve EAC overall 5-year survival to over 95%, identification of BE patients at risk to progress and subsequent inclusion in surveillance programs is highly important. However, only 5–10% of the EAC’s are detected in surveillance programs^14^. Thus, most EAC cases present as de novo cancers in advanced stage disease with poor prognosis^14^. BE by itself does not cause symptoms and is caused by gastro-esophageal reflux disease (GERD). BE patients are mostly diagnosed in case of accompanying symptoms of GERD for which they are examined by endoscopy^15^. To this end, the design of low-cost and efficient screening methods for the detection of BE in the general population is very desirable.

Blood biomarkers have become an essential tool for diagnosis and disease prognosis. They are simple, minimally invasive, economical, reliable, and rapidly obtainable measures of disease^16^. Therefore, the use of serologic biomarkers to detect BE is highly appealing since it could be applied for population screening programs to identify patients that need to undergo an endoscopic examination for the diagnosis of BE and should be included in surveillance programs. Consequently, this strategy would minimize the number of patients that will reach late EAC stage and improve the EAC survival rate. However, finding suitable serologic biomarkers for BE and EAC remains a major challenge. Due to methodological limitations, such as low sample size or lack of prospective validation studies, their introduction in the clinic is complicated^17^.

Bone morphogenetic proteins (BMPs) are multifunctional members of the TGFβ superfamily, which are involved in a wide range of biological processes^18,19^. BMP4 and its downstream targets have been reported to be present not only in the Barrett’s mucosa but also in the inflamed squamous epithelium as seen in GERD, while they have low expression in healthy squamous mucosa^20,21^. Proinflammatory factors, such as bile and gastric acids, lead to increased levels of BMP4 in the inflamed esophageal epithelium^20,22^. We presume that BMP4 may consequently activate stem cells, leading to initiation of gene transcription and enhancing the development of columnar epithelium^23^.

Several pieces of evidence make BMPs an attractive and suitable candidate as blood biomarkers for BE, since BMPs can be secreted extracellularly as soluble forms^19^. Previous studies have shown that BMP2, BMP4, BMP7, and BMP9 are present in the bloodstream and associated with several human diseases. For example, BMP2 is elevated in gastric cancer patients but is not associated with disease progression and development of a metastatic stage^24,25^. Similarly, BMP9 seems to have a role in fracture healing, as circulating levels of BMP9 were high in patients with fast fracture healing^26^. Furthermore, in patients with chronic kidney disease, circulating BMP2 plays an essential role in calcium deposition in vascular smooth muscles cells and vascular calcification^27^.

BMPs were not yet studied as potential blood biomarkers for epithelial metaplasia disease, such as in BE. Wang et al.^28^, described that not only BMP4, but also BMP2, BMP5 and BMP6 gene expression is upregulated in BE. Thus, in this study, we aimed to determine whether BMP2, BMP4, and BMP5 are detected in the plasma of BE patients. When compared to age and sex-matched control groups, we found that BMP2, BMP4, and BMP5 circulating levels were elevated in the BE patient group, with BMP2 and BMP5 showing a significant increase compared to the control group. However, in multivariate analysis, only BMP5 remained significant.

Our results suggest that the detection of BMP5 in plasma could represent a novel, fast, and economical diagnostic strategy to detect BE patients in the overall population. Future studies using a control group with confirmed healthy epithelium is required to validate and further develop the clinical translatability of this test.

## Results

### Gene expression analysis

**Figure 1:**
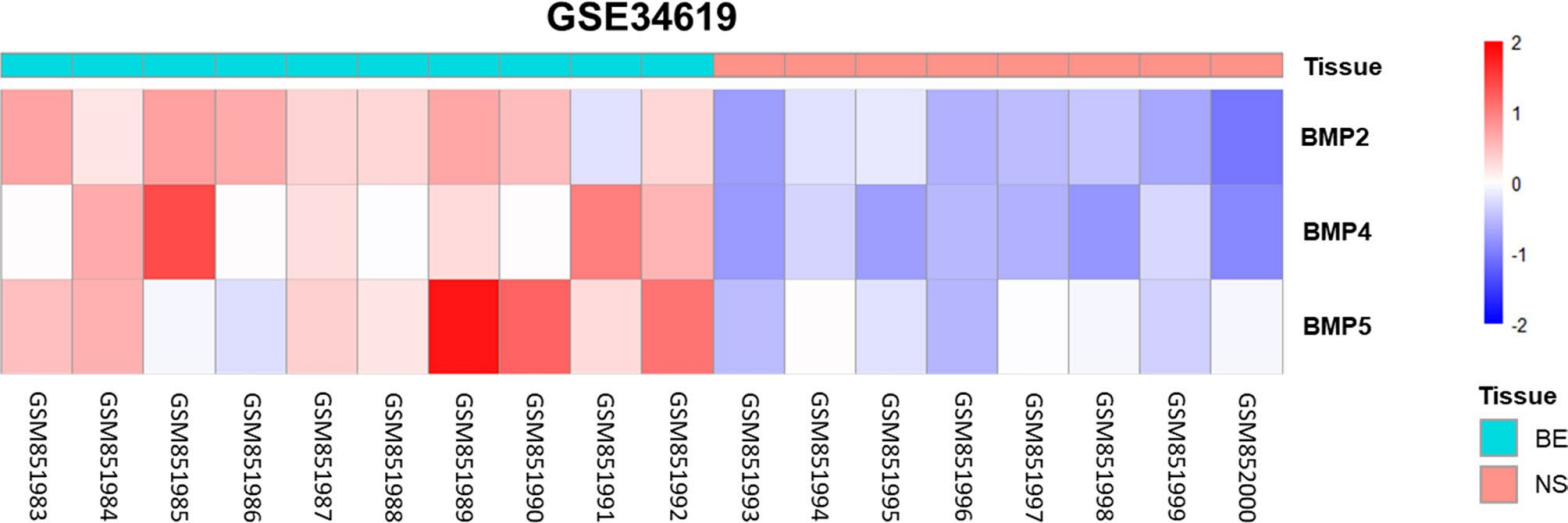
Heatmap showing expression of BMP2, BMP4 and BMP5 from Gene Expression Omnibus (accession number GSE34619) of patient biopsies of Barrett’s Esophagus and normal esophageal squamous epithelium. *B* Barrett’s Esophagus, *NS* normal squamous.

Analysis of BMP2, BMP4 and BMP5 gene expression of BE and normal esophageal squamous epithelium (NS), obtained from the Gene Expression Omnibus (accession number GSE34619), was performed (Fig. 1). Differential expression analysis showed that 6,324 probes were differentially expressed between BE and NS, after excluding unannotated probes. The log fold changes (based on geometric mean) were: BMP2 = 2.63, BMP4 = 2.84, and BMP5 = 2.19.

### Study population and patient characteristics

**Figure 2:**
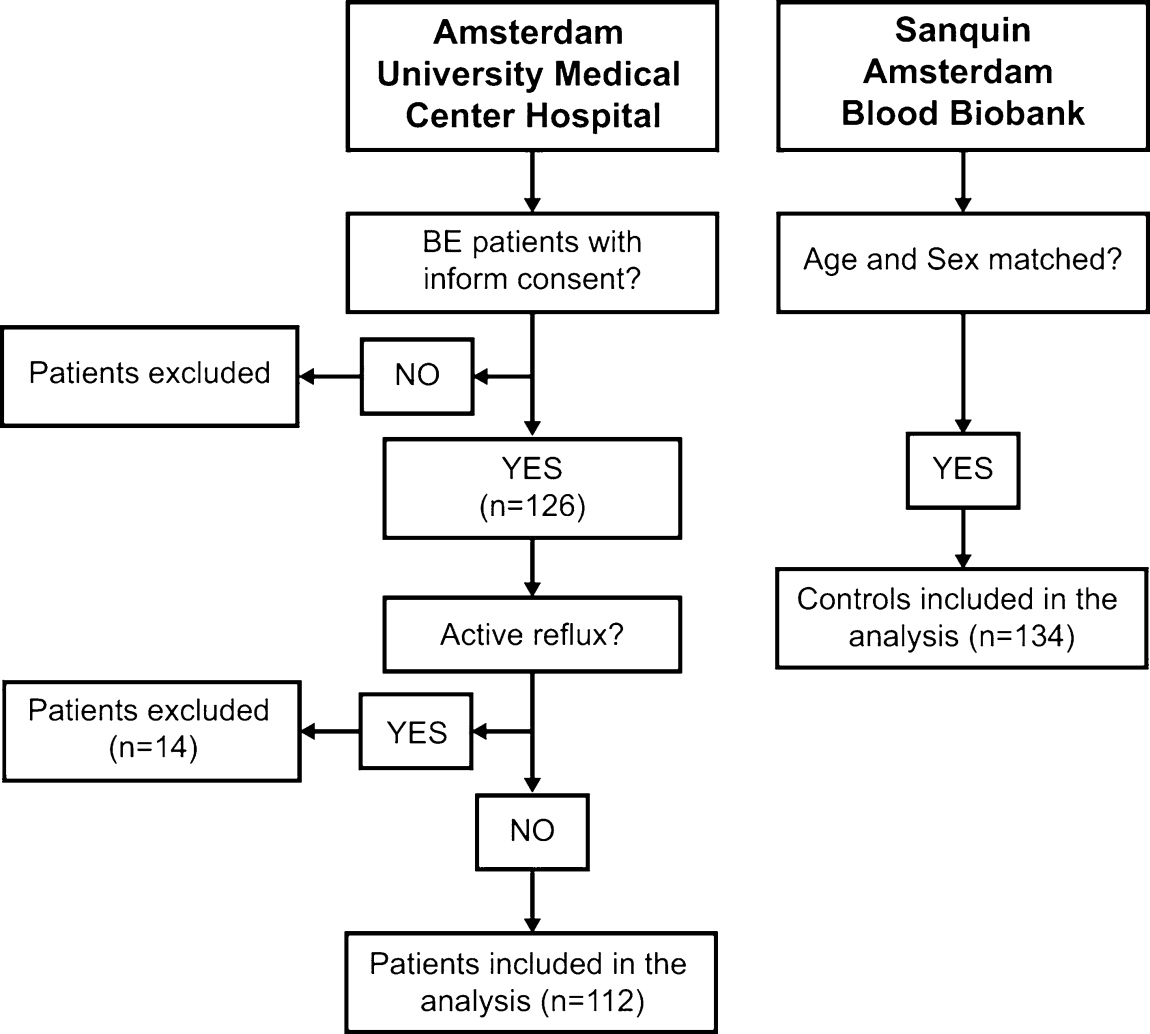
Flow diagram demonstrating patient and controls inclusion criteria.

In this study, a total of 246 participants were included. From these, 112 participants were BE patients from Amsterdam University Medical Centers, and 134 participants were sex- and age-matched controls from Amsterdam Blood Biobank, Sanquin. Since active reflux can confound results in terms of BMP expression, 14 BE patients with active reflux were excluded from the study (Fig. 2). The controls and patient demographics and clinical baseline data are shown in Table 1. The baseline characteristics between the two groups were evenly distributed in terms of age and sex. The median age in the BE patient group was 62 years (IQR 54–68) and in the control group 60 years (IQR 57–63), with a high male proportion in both two groups (BE group = 78.6%; control group = 81.3%). All BE cases included in the study group had endoscopically visible BE and intestinal-type of metaplasia, confirmed histologically by at least one experienced pathologist. Patients were endoscopically classified according to the Prague classification, presenting a median circumferential segment (C) of 1 (IQR 0–3) and a median maximum Barrett’s extent (M) of 3 (IQR 2–5). Additional clinical data was only available for the BE cases. Median BMI was 25.6 kg/m^2^ (IQR 23.8–29.1), where 43.1% patients had a normal weight, 36.7% were overweight, and 20.2% obese. Half of the patients were active or former smokers, and 44% were current alcohol consumers (> 2 units/day).

**Table 1:** Baseline characteristics of patients with Barrett’s esophagus (BE) and controls.

Cohort	Controls	Patients	*p* value
No. of subjects	134	112	
Age (years)	60 (57–63)	62 (54–68)	0.5
**Sex—n (%)**			
Male	109 (81.3%)	88 (78.6%)	0.7
**Median Barrett length (cm)**			
C	N.A	1 (0–3)	
M	NA	3(2–5)	
**Body mass index (kg/m**^**2**^**)**	N.A	25.6 (23.8–29.1)	
Normal	N.A	47 (43.1%)	
Overweight	N.A	40 (36.7%)	
Obese	NA	22 (20.2%)	
**Smoking**			
Curent smoker	N.A	15 (13.8%)	
Former smoker	N.A	42 (38.5%)	
Never smoker	N.A	52 (47.7%)	
**Alcohol consumption**			
Yes	N.A	48 (44.0%)	

Data are median (IQR) or frequency (%).

*C* circumferential segment, *M* maximum Barrett’s extent, *IQR* interquartile range, *ns* not significant, *N.A.* not available.

### BMP levels among study groups

**Figure 3:**
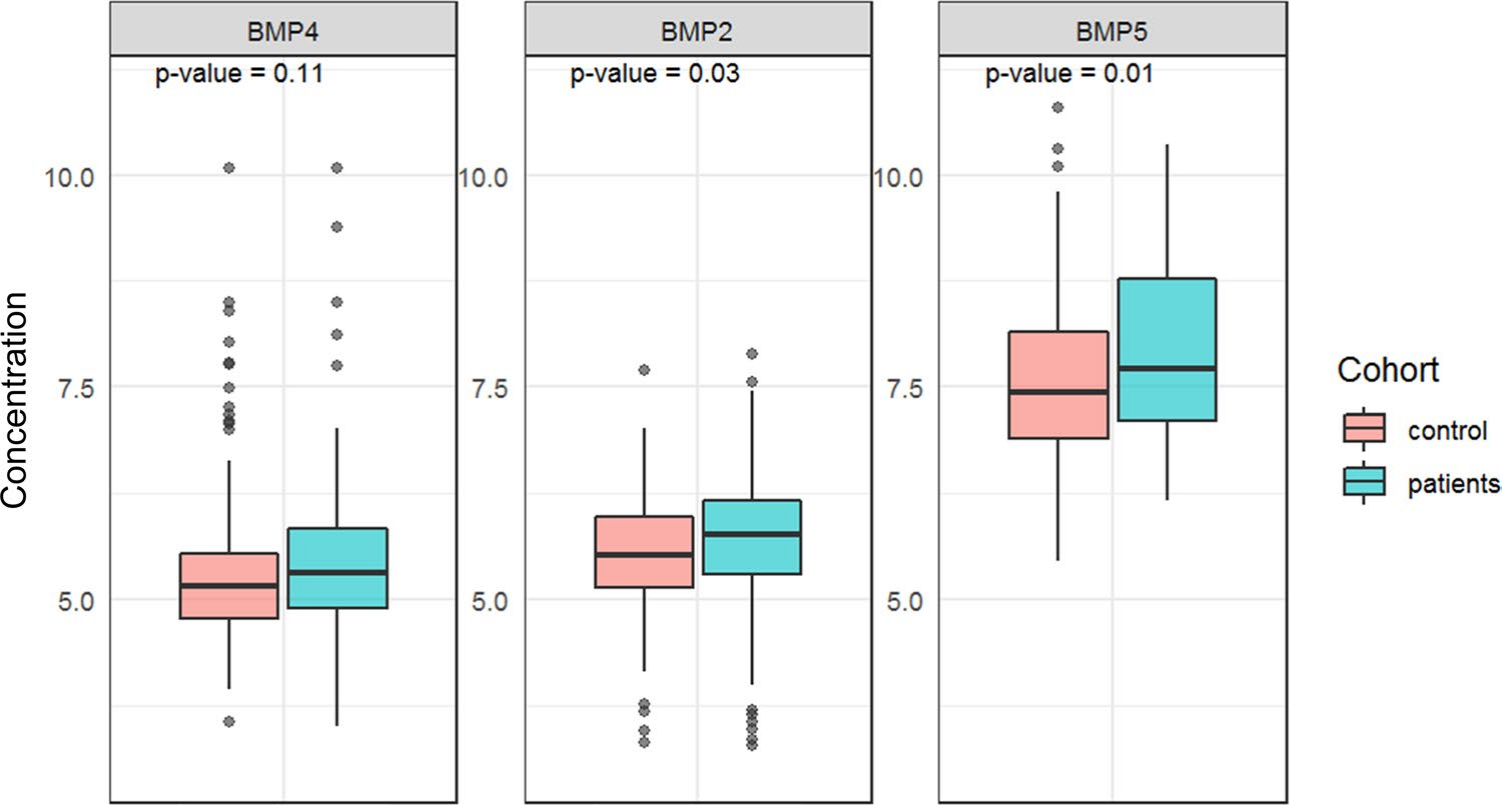
Box plots of the distribution of log-transformed protein biomarkers by BE patients (blue) and controls (red). Each panel corresponds to a different biomarker. Horizontal lines represent the median and boxes to the 25th–75th percentiles.

The plasma levels of the different BMPs were measured using the ELISA sandwich method in combination with MSD technology. Figure 3 shows the box plots of the distribution of the BMP levels (log-transformed) for patients and controls. Among the analysis, the maximum percentage of imputed values was 2.85% (BMP2). The median concentration of the different biomarkers was generally higher in the patient group. Median concentration levels of BMP5 [2,251.9 pg/mL (IQR 1,210.4–6,865.1 pg/mL)] and BMP2 [317.9 pg/mL (IQR 198.7–475.8 pg/mL)] were significantly higher in patients than controls [BMP5 (1701 pg/mL (IQR 990.9–3,449 pg/mL), *p* value = 0.01] and BMP2 [248.8 pg/mL (IQR 171.1–393.3 pg/mL), *p* value = 0.03]. Although the median concentration of BMP4 was higher in the patient group [204.1 pg/mL (IQR 133.1–339.3 pg/mL)], the difference was not statistically significant [BMP4 control group: 173.5 pg/mL (IQR 117.1–252.5 pg/mL), *p* value = 0.11].

### BMP5 is associated with an increased risk of having BE

Initially, we performed univariate logistic regression to assess the effect of each BMP on the risk of having BE separately (Table 2). The higher concentration of BMP5 was significantly associated with an increased risk of BE. The odds ratio (OR) was 1.44 (95% CI 1.12–1.87; *p* value = 0.01) per pg/mL increase in log-transformed BMP5. Although higher concentrations of BMP4 [OR = 1.11 (95% CI 0.86–1.43; *p* value = 0.44)] and BMP2 [OR = 1.34 (95% CI 0.98–1.87; *p* value = 0.07)] were observed in association with risk of having BE as well, these associations were not significant. Additionally, we performed multivariate logistic regression to assess the effect of all BMPs on BE. The OR for having BE decreased for BMP4 (OR = 0.83, 95% CI 0.58–1.15) and BMP2 (OR = 1.23, 95% CI 0.82–1.86), while BMP5 slightly increased (OR = 1.47, 95% CI 1.09–2.02) (Table 2). When adjusting the data for the effect of age and sex, the OR per log-transformed for BMP4 and BMP2 remained the same, while BMP5 improved (OR = 1.49, 95% CI 1.10–2.05) (Table 3). In all logistic regression analyses, the high concentration levels of BMP5 were significantly associated with the increased risk of the presence of BE. Our data suggest that per pg/mL increase in the natural logarithm of plasma concentration of BMP5, the odds of having BE increases by 50%.

**Table 2:** Univariate and multivariate logistic regression model for the effect of different BMPs on the development of BE.

Biomarker	Univariate			Multivariate		
	OR	95% CI	*p* value	OR	95% CI	*p* value
BMP4	1.11	0.86–1.43	0.44	0.83	0.58–1.15	0.26
BMP2	1.34	0.98–1.87	0.07	1.23	0.82–1.86	0.31
BMP5	1.44	1.12–1.87	0.01**	1.47	1.09–2.02	0.01**

Multivariate analysis adjusted for BMP4, BMP2, and BMP5. All proteins have been natural log-transformed.

*OR* (odd ratio) for developing BE per pg/mL increase in the logarithm of each protein, *CI* confidence interval.

**p value < 0.01.

**Table 3:** Multivariate logistic regression model for the effect of different BMPs on the development of BE.

Variable	Multivariate		
	OR	95% CI	*p* value
BMP4	0.83	0.58–1.15	0.26
BMP2	1.23	0.82–1.86	0.31
BMP5	1.49	1.10–2.05	0.01**
Age	1.02	0.98–1.05	0.33
Sex: male	0.85	0.45–1.63	0.63

Analysis adjusted for BMP4, BMP2, BMP5, age and sex. All data have been natural log-transformed.

*OR* odd ratio, *CI* confidence interval.

**p value < 0.01.

### Effect of risk factors on BMP5 among BE patients

Besides active reflux, increased age (50 years or older), male sex, other risk factors associated with the presence of BE includes high body mass index (BMI), Caucasian race, smoking, and a family history of BE or EAC^29,30^.

Since BMP5 was the most promising BMP associated with the risk of developing BE, we subsequently performed multivariate analyses of different known risk factors associated with BE together with plasma levels of BMP5 (Table 4). No significant association was observed between BMP5 levels and different patient characteristics. Active smokers showed a weak negative association (β = − 0.38; *p* value = 0.28), while in patients that consumed alcohol, we observed a weak positive association (β = 0.17; *p* value = 0.45) with the levels of BMP5.

**Table 4:** Association between BMP5 and risk factors within BE patients.

Variable	Estimate (*β*)	*p* value
Male	− 0.09	0.72
Age	− 0.01	0.38
BMI	− 0.02	0.44
M	0.06	0.58
C	− 0.07	0.51
Active smoker	− 0.38	0.28
Former smoker	0.02	0.94
Active alcohol intake	0.17	0.45

*BMI* body mass index; *C* circumferential segment, *M* maximum Barrett’s extent, *OR* odd ratio, CI confidence interval.

**p value < 0.01.

## Discussion

The incidence of EAC is increasing, while the majority of patients with BE, a significant risk factor for EAC, are not diagnosed^12^. BE patients that are not included in periodic endoscopic surveillance are at risk of developing the advanced stage EAC which is associated with poor outcomes. One way to prevent advanced stage EAC is to detect BE patients in population screening programs. For such programs, efficient, non-invasive and cost-effective tools are required. A potential attractive approach is the use of blood biomarkers as a screening tool for BE.

We and others have previously described the role of BMP4 in BE metaplasia^20,21,31,32^. Therefore, the use of BMP4 as a candidate circulating biomarker for BE is of great interest. To our knowledge, this is the first study analysing circulating BMPs as a potential biomarker to be used as a screening tool for BE patients. Over the past years, several studies have identified blood-based biomarker candidates^33^. Some promising studies focused on the determination of single microRNAs or circulating glycoproteins as biomarker candidates^34,35^. However, most of the studies described in the literature have limited sample size, no age and sex-matched controls, and lack of validation studies, which limits their establishment in the clinic.

BMPs can be secreted as soluble forms in intracellular spaces such as in the esophageal mucosa and detected in the bloodstream^19^. In the present study, we were able to detect BMP4, BMP2, and BMP5 in the plasma of BE patients, using the MSD detection technology. The MSD detection platform contributed to higher sensitivity and greater range to detect our analytes of choice with a stronger and specific signal when compared with the conventional sandwich ELISA detection method (data not shown). We observed elevated concentration levels of all three BMPs in the patient group when compared with the control group, with a significant difference for BMP2 and BMP5. After multivariate analysis BMP5 proved to remain significant and was the strongest risk factor.

BMP5 has an important role during embryonic musculoskeletal development and has been associated with different human diseases^36^. In the gastrointestinal system, BMP5 has been described as one of the key proteins to maintain colon crypt stem cell niche in both normal and neoplastic conditions^37^. Furthermore, BMP5 has been linked as an important tumour suppressor in human colorectal cancer^38^ and may play an important role in esophageal tumorigenesis in human esophageal squamous cell carcinoma^39^. However, the levels and role of BMP5 can differ among tumours. Additionally, BMP5 together with BMP4 have a dualistic role in some pancreatic cancer cell lines, acting simultaneously as anti-proliferative factors inhibiting cell growth as well as pro-metastatic factors through stimulation of cell migration and invasion^40^. Although BMP5 has been seen in osseous metaplasia in colon cancer patients, in esophageal metaplastic diseases, such as BE, the role of BMP5 has not been described^41^. As far as we know, this is the first association of BMP5 with BE. To better understand the role of BMP5 in BE, further molecular studies should be performed.

Upregulated levels of BMP4 at the protein and mRNA level in BE biopsy samples compared with squamous biopsies have been previously described^20,42^. In this case–control study, the levels of circulating BMP4 were surprisingly lower when compared with BMP2 and BMP5, and presented no significant difference between BE patients and controls. One possible explanation is that BMP4 is less secreted compared to the other BMPs or that the secreted form is immobilized or masked through binding to other proteins, such as fibrinogen, which is part of plasma composition and known to form complexes with different growth factors such as BMPs^43^. The relatively lower circulating levels of BMP4 in BE patients might also be the result of their recruitment to the inflamed esophageal epithelium. Therefore, circulating BMP4 may not reflect the alterations occurring in the tissue. A similar situation was observed for other proteins. For instance, cathepsin E mRNA is significantly higher in BE tissue compared to its expression in normal esophageal tissue, yet no significant difference was observed in its serum levels of BE patients and controls^44^. Additionally, Vrielin et al.^45^, showed that the mRNA levels of the IGF-system components were significantly higher in the colorectum tissue, while serum IGF-I/-II concentration was not. These observations suggest that local and circulating protein levels can be differently regulated.

For our study, we were able to collect blood samples from a large number of BE patients. Our control samples were obtained from the same geographical region as the BE patients. One limitation of our study, due to privacy matters, is that only information regarding age and sex was provided for the control samples. Therefore, patients with diagnosed or undiagnosed BE are potentially included in the control group. In the geographical region, from which patients were included, it is estimated that 10–20% of the persons have GERD, of which 5–15% may have BE^9^. Moreover, this might even be an underestimation of the percentage of BE patients in the control group, as the prevalence of BE is even higher in males of 50 years, and older^46^. Since our control group mainly consisted of middle-aged males, it is most likely that a subset of the controls had BE or active GERD. This limitation probably confounded our results and could explain the high levels of circulating BMPs in specific individuals within the control group. From our patient group we obtained more detailed information about the BE risk factors. When associating the BMP5 levels with these risk factors, none of them was significantly associated with increased BMP5 plasma levels. Thus, BMP5 seems to be an independent risk factor for BE.

In summary, we found that BMP2, BMP4, and BMP5 were higher in the blood of BE patients when compared with sex and age-matched controls. Plasma levels of BMP5 were significantly higher and associated with the risk of BE. In addition, large cohort studies of blood sampling in conjunction with endoscopic analyses in asymptomatic participants would be required to determine whether circulating BMP5 might represent a tool to detect asymptomatic BE. Thus, our study, represents an important stepping stone for the use of circulating BMP5 as a diagnostic test. However, our results need to be validated in larger independent cohorts before implementation in population screening programs.

## Methods

### Study populations

A total of 112 BE patients were included in our study. All included patients were on long-term proton pump inhibition therapy and had a confirmed diagnosis of BE by endoscopy and histology. BE patients with active reflux were excluded from the study (n = 14). The median age of all patients included was 62 years (range, 54–68 years), and 78.6% of the patients were male.

Peripheral blood samples were collected during the routine surveillance program at the Gastroenterology and Hepatology Department, Amsterdam University Medical Center. Agreed written inform consent was obtained from all patients to participate in this study. The ethical board of the Gastroenterology and Hepatology Department at the Amsterdam University Medical Center granted approval, and the study was conducted according our institution ethical guidelines and the Helsinki Declaration.

Control blood samples of 134 individuals were purchased from Sanquin Blood Supply (The Netherlands). Age and sex in the control group were matched with the patient group, with a median age of 60 years (range 57–63 years) and 81.3% of males.

### Blood sample collection and storage

Blood samples from all human participants were collected into 5 mL vacutainer tubes (BD Vacutainer) with Ethylenediaminetetraacetic acid as an anticoagulant. Samples were separated within 2 h of the collection while kept on ice. Samples were spun at 1,500 rpm for 15 min at − 4 °C, aliquoted and stored at − 80 °C until further analysis.

### Meso Scale Discovery (MSD) electrochemiluminescence detection

We developed an MSD-based screening platform to quantify circulating BMP2, BMP4, and BMP5. The MSD platform used in this study was a 96-well sandwich immunoassay that incorporated electrochemiluminescence as the basis for detection. A combination of capture and detection antibodies was initially screened for optimal antibody orientation for the sandwich immunoassay to achieve better dynamic range, sensitivity, and specificity. Ultimately, the antibody pairs of the R&D DuoSet ELISA for the respective BMPs were used (BMP4: Cat. No. DY314; BMP2: Cat. No. DY355; BMP5: Cat. No. DY615B; all R&D Systems). The protocol was adapted and performed following the MSD assay workflow steps. Briefly, a quickplex 96-well high-bind plate (Cat. No. L55XB-3, MSD) was coated with 30 µL of coating antibody overnight. The plate was washed four times with washing buffer (Phosphate-Buffered Saline (PBS) with 0.05% Tween-20) and blocked with blocking solution [PBS with 5% Blocker A (Cat. No. R93BA-4, MSD)] and incubated for 1 h. Afterward, plates were washed, and 25 µL of sample or standard were added to the wells and incubated for 2 h at room temperature (RT) with shaking. Samples were discarded, and wells washed. Next, 25 µL of the detection antibody was added and incubated for 2 h at RT with shaking. Subsequently, the detection antibody was discarded, and plates washed. 25 µL of Streptavidin-SulfoTAG (Cat. No. R32AD-5, MSD) was added to the wells and incubated for 1 h at RT with shaking. As a final step, 150 µL of MSD reading buffer (2 × concentrated; Cat. No. R92TC-1, MSD) was added, and the plate was read using the MESO QuickPlex SQ 120 imager and analysed using MSDs Discovery Workbench Software, version 4.0.

### Bioinformatics analysis

Gene expression profiles of Barrett’s esophagus and esophageal squamous epithelium were downloaded from the Gene Expression Omnibus (accession number GSE34619). Data had been generated using Affymetrix Human Gene 1.0 ST array and was already pre-processed. 10 profiles from Barrett’s biopsies were compared to 8 profiles from adjacent squamous epithelium. Chip annotation GPL6244 was used for annotation of the probe sets. Differential expression analysis was performed using the Limma package. Probesets with multiple-test corrected *p* value < 0.05 were considered significantly differentially expressed. The z-scores of the genes of interest were visualized in a heatmap.

### Statistical analysis

The concentration of the different biomarkers was imputed when measurements were out of range of the calibration curve (either too low [bellow lower limit of detection (< LOD)] or too high) based on a maximum likelihood estimation procedure^47^. Imputation is a statistic strategy that produces unbiased estimations to minimize extreme data values. For the imputation of samples < LOD, we used the empirical LOD across all plates as the upper bound. For imputation of samples with a concentration exceeding the calibration curve, we used a value of twice the highest observed concentration that was not out of range as the upper bound. Categorical variables were expressed as counts and corresponding percentages, continuous data were presented as the median and interquartile range (IQR).

To reduce the effect of extreme values, log-transformation of the different proteins has been used in all analyses. The concentration of the biomarkers between BE patients and the control group were first compared using the Mann–Whitney test. Additionally, to assess the effect of protein biomarkers on developing BE, univariate and multivariate logistic regression was performed. The analysis was performed in statistical software R (version 3.5.1). *p* values < 0.05 were considered statistically significant.
